# Under-Resourced or Overloaded? Rethinking Working Memory Deficits in Developmental Language Disorder

**DOI:** 10.1037/rev0000338

**Published:** 2022-04-28

**Authors:** Samuel David Jones, Gert Westermann

**Affiliations:** 1 Department of Psychology, Fylde College, Lancaster University

**Keywords:** developmental language disorder, spoken word recognition, word learning, convolutional neural network, manifold geometry

## Abstract

Dominant theoretical accounts of developmental language disorder (DLD) commonly invoke working memory capacity limitations. In the current report, we present an alternative view: That working memory in DLD is not under-resourced but overloaded due to operating on speech representations with low discriminability. This account is developed through computational simulations involving deep convolutional neural networks trained on spoken word spectrograms in which information is either retained to mimic typical development or degraded to mimic the auditory processing deficits identified among some children with DLD. We assess not only spoken word recognition accuracy and predictive probability and entropy (i.e., predictive distribution spread), but also use mean-field-theory based manifold analysis to assess; (a) internal speech representation dimensionality and (b) classification capacity, a measure of the networks’ ability to isolate any given internal speech representation that is used as a proxy for attentional control. We show that instantiating a low-level auditory processing deficit results in the formation of internal speech representations with atypically high dimensionality, and that classification capacity is exhausted due to low representation separability. These representation and control deficits underpin not only lower performance accuracy but also greater uncertainty even when making accurate predictions in a simulated spoken word recognition task (i.e., predictive distributions with low maximum probability and high entropy), which replicates the response delays and word finding difficulties often seen in DLD. Overall, these simulations demonstrate a theoretical account of speech representation and processing deficits in DLD in which working memory capacity limitations play no causal role.

Learning language is a central aspect of child development and is often mastered with astonishing ease despite the complexity of language and a lack of direct instruction. Nevertheless, not all children succeed equally in acquiring language. In developmental language disorder (DLD), deficits in spoken language comprehension and production severe enough to affect the child’s wellbeing are observed despite no obvious biomedical cause (Bishop et al., 2016). Although DLD is widespread, affecting approximately 7.5% of English-speaking children (Norbury et al., 2016), much remains unknown about the causal mechanisms underlying this condition.

A dominant feature of existing causal accounts of DLD is an emphasis on the role of working memory. Apparently uniformly, research in this area has taken lead from Baddeley and Hitch’s (1974) multicomponent model, which comprises a central executive that attends to and manipulates information stored temporarily in one of three modality-specific buffer systems; the visuospatial sketchpad, the episodic buffer, and the phonological loop. Research into the causal origins of DLD has focused principally on the role of the phonological loop in the temporary retention of speech signals, and the role of the central executive in retrieving and manipulating speech signals.^1^

Performance deficits in tasks thought to test the integrity of the working memory system are perhaps the most consistent finding in DLD research. Children with DLD commonly score poorly, for instance, in the nonword repetition task, in which participants are required to repeat recently heard auditory stimuli such as *doppelate*, *hampent*, or *glistering*, a task commonly held to tap phonological loop capacity (see Vance, 2008, for review). Performance deficits in the nonword repetition task and related paradigms among children with DLD underpin the consensus view that capacity limitations in both the central executive and phonological loop subsystems of working memory play a causal role in these children’s language difficulties, directly obstructing the temporary retention, retrieval, and manipulation of speech signals, and resulting in degraded long-term speech representations during learning (Archibald & Gathercole, 2006a; Archibald & Harder Griebeling, 2016; Delage & Durrleman, 2018; Delage & Frauenfelder, 2020; Durrleman & Delage, 2016; Ellis Weismer et al., 2017; Jakubowicz, 2011; Montgomery, 1995, 2003; Montgomery et al., 2019; Zebib et al., 2020; cf. Howard & van der Lely, 1995; van der Lely & Howard, 1993; see also Kail, 1994, for an account citing generalized slowing rather than specific working memory capacity deficits).

Yet, despite the dominance of the causal view of working memory capacity limitations in DLD, much of the evidence cited in support of this position is correlational. A child might show a nonword repetition task performance deficit alongside a deficit in vocabulary size or sentence comprehension, for instance, and a causal association between a hypothesized underlying working memory capacity limitation and relatively poor language skills is inferred on this basis (e.g., Montgomery, 1995; note that more recent studies assess such correlations using more advanced methods, including mediation and cross-lagged designs, e.g., Boerma & Blom, 2020). Alternatively, some studies have sought to identify domain general working memory capacity deficits in children with DLD, for instance deficits implicating both verbal and visual working memory subsystems; the former measured using tasks such as nonword repetition and the latter measured using visual pattern recognition and spatial span tasks (Archibald & Gathercole, 2006b; Bavin et al., 2005; Henry & Botting, 2017). Here, the identification of domain general deficits is argued to bolster the view that working memory capacity limitations play a primary role in language impairment, ensuring that performance deficits are not simply an epiphenomenon of shortfalls in long-term language knowledge. However, this position remains contentious, with some studies reporting no evidence of visual working memory task performance deficits among children affected by DLD, a finding lending apparent support to the claim that the underlying problem is specific to the verbal working memory system (Archibald & Gathercole, 2006b).

Seemingly stronger evidence for a causal association between working memory capacity limitations and language impairment comes from studies reporting nonword repetition task performance deficits in individuals whose language problems have been resolved through intervention (Bishop et al., 1996). This pattern would apparently not be expected if working memory task performance deficits purely reflected insufficient long-term language knowledge. Yet, as these authors acknowledge, alongside others (e.g., Coady & Evans, 2008; Melby-Lervåg et al., 2012), the once common interpretation of nonword repetition task performance as a relatively pure measure of working memory capacity, specifically phonological loop capacity, is misplaced, as nonword repetition implicates a wide range of skills including auditory perception, speech planning, and articulation. While this more nuanced interpretation of what is measured in the nonword repetition task and closely related paradigms in no way challenges the validity of using such measures to identify individuals with existing language impairment, or potentially with a history of language impairment, it does undermine the view that what we are detecting in administering such tasks is a pure working memory capacity limitation. The picture is complex, and deficits in, for instance, nonword repetition task performance despite largely resolved language difficulties may reflect residual deficits in any number of skills.

In our view, the causal account of working memory capacity limitations in DLD remains dominant because the field lacks a cohesive alternative. This has important practical implications. An alternative theoretical framework in which working memory capacity limitations do not feature may not only provide a more compelling explanation of the behavioral data at hand, but it may also entail different approaches to language support. Evidence interpreted as signaling a causal association between limited working memory capacity and language deficits has motivated the development of commercial packages claiming to improve working memory capacity and in doing so boost language and educational outcomes (e.g., *Jungle Memory*; Alloway et al., 2013). However, if working memory capacity limitations are not a major underlying cause of language deficits then interventions may need to focus on a different aspect of cognition or language processing in order to achieve substantial and lasting effects. It is important to reiterate that working memory task performance remains one of the best predictors of language impairment (Bishop et al., 1996; Girbau, 2016; Kalnak et al., 2014), and that the validity of using such paradigms to statistically identify individuals at risk of language problems is not in question. What is in question, is whether apparent working memory capacity limitations are the cause, rather than consequence, of the language learning and processing difficulties seen among children with DLD.

## Rethinking Working Memory Capacity Deficits in DLD

The view developed in this report is that working memory capacity limitations are the consequence rather than cause of children’s language difficulties. Crucial to this account is the notion of a capacity and performance trade-off. It is uncontroversial that long-term knowledge affects working memory task performance (Vance, 2008). In both typically and atypically developing populations, performance is seen to decline (e.g., in terms of the length of speech segments that can be accurately recalled) when individuals are presented with unfamiliar stimuli, as seen in word-likeness effects (i.e., phonologically anomalous nonwords are harder to repeat; Gathercole, 1995; Van Bon & Van Der Pijl, 1997) and in responses to noisy stimuli (Marrone et al., 2015). The idea of a capacity and performance trade-off suggests that this drop in performance emerges due to working memory being overloaded as a result of heightened processing demands. In contrast, faced with broadly familiar, nonnoisy stimuli, processing resources are not under pressure and so more information can be retained.

One possibility, then, is that performance deficits widely attributed to working memory capacity limitations among children with DLD instead reflect heightened processing demands resulting from deficits in long-term language knowledge, including poorly configured long-term speech representations (Kan & Windsor, 2010). This issue may be masked by the fact that the stimuli presented to children with and without DLD in working memory tasks are usually matched; for example, stimuli are either all clean or all noisy across groups. Yet, if a child with DLD has deficient speech encoding ability then their experience of any given stimulus will be very different to that of a same-age child without language impairment, increasing processing demands for this child and exhausting cognitive resources that could be allocated to storage capacity. Rather than fixed, group-level disparities in working memory capacity, then, the difference between children with and without DLD may resemble the ostensible capacity discrepancies that can be seen in a single typically developing child who is presented with noisy and then clean stimuli, and who retains more information in the second instance. Children with DLD may not be under-resourced in terms of their working memory capacity as the consensus holds but may instead be overloaded by heightened processing demands given poorly configured long-term speech representations. Though relatively unexplored, limited evidence in support of this position includes an apparent absence of working memory task performance deficits between children with DLD and control children matched on long-term language knowledge (van der Lely & Howard, 1993).

This view of working memory capacity limitations as the consequence rather than cause of language difficulties aligns well with contemporary working memory frameworks that seek to de-emphasize the role of functionally discrete, modality-specific buffers, such as the phonological loop, in favor of a relatively parsimonious characterization of working memory in terms of activated long-term memory plus attention (Adams et al., 2018; Chai et al., 2018; Cowan, 1995; D’Esposito & Postle, 2015; McElree, 2006; Oberauer, 2013, 2019; Wilhelm et al., 2013). The so-called state-based framework of working memory, popularized through Cowan’s embedded-processes model (Cowan, 1995, 1999) and later notably developed by McElree (2006) and Oberauer (2013), is outlined by Adams et al. (2018) as follows:

Information comes in from the environment through a very brief sensory store, activating features in long-term memory corresponding to the sensory properties of the incoming information and its coding: phonological, orthographic, visual, and other simple features from the senses. ... The activated features from long-term memory, including any newly formed memories, along with the current focus of attention, together comprise the working memory system. (p. 345)

For some, the state-based working memory framework represents simply a difference in terminology and research focus (e.g., a heightened interest in the role of attention versus modality-specific processing), rather than a clear theoretical break with the earlier multicomponent model that continues to dominate DLD research (Baddeley, 2012). Yet, in our view, the implications of the state-based framework for theory building in DLD are significant. Crucially, the framework encourages a theoretical shift in the locus of impairment from a shortfall in a functionally discrete buffer system (i.e., the phonological loop), to deficits in the quality of long-term speech representations, and the associated efficacy with which such representations become activated in response to features of the speech environment and are therefore amenable to forming the focus of attention. As Oberauer (2019) has argued, it is essential that long-term representations are encoded in a manner supporting efficient activation and the effective deployment of attention. In this report, we argue that atypical long-term speech representation encoding and activation in DLD result in attention being overloaded in the absence of any fundamental capacity limitation.

The challenge for mechanistic accounts arguing that apparent working memory capacity limitations are the consequence of shortfalls in long-term language knowledge is, of course, to explain how and why speech encoding is deficient without appealing to a primary working memory capacity bottleneck. Along these lines, computational modeling of variance in nonword repetition and span task performance among typically developing individuals has appealed to the notions of input frequency and regularity (Jones, 2016; Jones et al., 2007, 2008, 2020; MacDonald & Christiansen, 2002). Here, the idea is that the ability of an artificial neural network to accurately process any given speech sequence relates directly to the quality of the network’s established, analogous representations, which is higher when the relevant input previously received is frequent and structurally consistent. In one landmark study, for instance, MacDonald and Christiansen (2002) showed, in neural networks without functionally discrete working memory systems, that performance deficits analogous to those attributed to verbal working memory capacity limitations by Just and Carpenter (1992) diminished with each cycle of training. This indicates that a separate buffer system which hypothetically varies in capacity between individuals (e.g., a phonological loop) is not required to explain variance in task performance; variance in the frequency of stimulus exposure and therefore the quality of long-term encodings (i.e., more frequently encountered, regular stimuli are better encoded) can parsimoniously account for the data at hand.

The long-term encoding benefits of high frequency and regularity of exposure clearly boost performance for certain stimuli in working memory tasks, and may more broadly explain why working memory capacity appears to increase during infancy and childhood (Jones et al., 2020). Simply, as implicit in the state-based framework of working memory, task performance may improve as children become increasingly adept at deploying their mounting long-term language knowledge in the moment, not, as is commonly argued, because of developmental capacity increases that are independent of the quality of long-term representations (Gathercole et al., 2004). Yet, a notion of language familiarity grounded in the degree and quality of language exposure alone is unsatisfactory as an explanation of the language profiles seen in DLD. Evidence for this comes not least from twin studies, which show that dizygotic twins, who are no more genetically similar than regular siblings but largely share a language environment, can be differentially affected by DLD; an observation indicating a genetic component to this disorder (Bishop, 2006). Clearly, then, if we are to better understand how a working memory capacity overload might emerge as a consequence of atypical speech representation, it is necessary to go beyond the notions of input frequency and regularity alone to consider shortfalls in the child’s ability to encode speech information from their environment.

Auditory processing deficits commonly reported among children with DLD provide a credible starting point for this form of inquiry. While initially cast as a temporal processing issue, that is, that some children affected by DLD have difficulty discriminating rapidly occurring changes in pure tone—a view developed through the work of Paula Tallal and colleagues (e.g., Merzenich et al., 1996; Tallal et al., 1996)—subsequent studies suggest that the problem may instead lie in frequency discrimination, aside from the speed of stimulus presentation (Bishop et al., 1999; Bishop & McArthur, 2005; McArthur & Bishop, 2005). For instance, in an electroencephalography (EEG) study incorporating an oddball paradigm, Bishop and McArthur (2005) found group deficits among children with DLD in the ability to identify, through button pressing, differences in frequency between 600 Hz and 700 Hz that were independent of the rate of stimulus presentation. Importantly, not only did children with DLD in this study score poorly on behavioral measures (i.e., in their rate of accurate button presses in response to tone sequences), but EEG analysis also highlighted atypical waveforms even when these children made accurate responses. This result suggests that atypical frequency processing may be at play even when performance in a frequency discrimination task, such as those widely used in the initial screening phase of behavioral assessments involving children with DLD, is apparently standard. Frequency discrimination deficits may, therefore, be more widespread than thought in this population.

It may appear reasonable to assume a causal association between low-level frequency discrimination deficits and the deficits in higher-order speech representation and retrieval that characterize DLD. Children affected by DLD commonly require more exposures to a spoken word than control children in order to encode similar levels of phonological detail (Gray, 2003), for instance, and are often slower and less accurate than age-matched peers when retrieving words and naming known objects (Kambanaros et al., 2015; Messer & Dockrell, 2006), when determining whether an auditory stimulus is a known word or nonword (Jones & Brandt, 2018), when fixing their gaze to a named visual stimulus (McMurray et al., 2019), when identifying words from clipped auditory segments (Montgomery, 1999), when identifying mispronunciations (Alt & Suddarth, 2012), and, as previously discussed, when repeating nonwords (Bishop et al., 1996). These performance deficits between children with and without DLD may be explained in terms of lower familiarity with the target stimuli among children with DLD, which is itself a function of the quality of the speech representations that these children have formed. Evans et al. (2018), for instance, found no spoken word recognition accuracy discrepancies between children with and without DLD in a gating paradigm in which target word knowledge was controlled. Nevertheless, whether and how such higher-order speech representation deficits relate to underlying abnormalities in frequency discrimination remains unclear, and assuming a casual association here remains controversial in lieu of a satisfactory linking hypothesis (Bishop & McArthur, 2005; McArthur & Bishop, 2005).

Furthermore, despite a wealth of behavioral evidence pointing to speech representation deficits in children with DLD (e.g., the aforementioned evidence from the naming, mispronunciation, identification, and nonword repetition tasks), a precise account of the form that such deficits take remains elusive, with existing research restricted to verbal descriptions of task performance being impeded due to the *fuzziness*, *imprecision*, or *indistinctiveness* of underlying long-term speech representations (Alt & Suddarth, 2012; Claessen et al., 2009; Claessen & Leitão, 2012; Maillart et al., 2004). In the present study, we aim to address each of these gaps in current understanding: First, by demonstrating a causal association between auditory processing deficits and deficits in higher-order speech representation and retrieval, and second by providing a precise, computational account of the nature of speech representation and retrieval deficits in DLD that we believe provides an essential counterpart to existing verbal theories. Our aim is to demonstrate how auditory–perceptual deficits can explain deficits in long-term speech representation, which in turn explain communication deficits by way of attention being overloaded, rather than by way of working memory capacity limitations that are independent of the quality of long-term speech encodings.

## Speech Processing From Cochlea to Cortex

The theoretical account presented in this report is informed by the manifold untangling framework developed in visual neuroscience (DiCarlo & Cox, 2007) and recently applied in studies of speech processing and representation (Kell et al., 2018; Stephenson et al., 2020). Manifold untangling describes an integrated theoretical and computational approach to studying neurobiological processes. In this section, our focus is on theory, specifically how manifold untangling shapes the view of speech perception and processing in DLD that we have outlined. Details of the computational implementation of this framework are discussed in the *Method* section.

**Figure 1 fig1:**
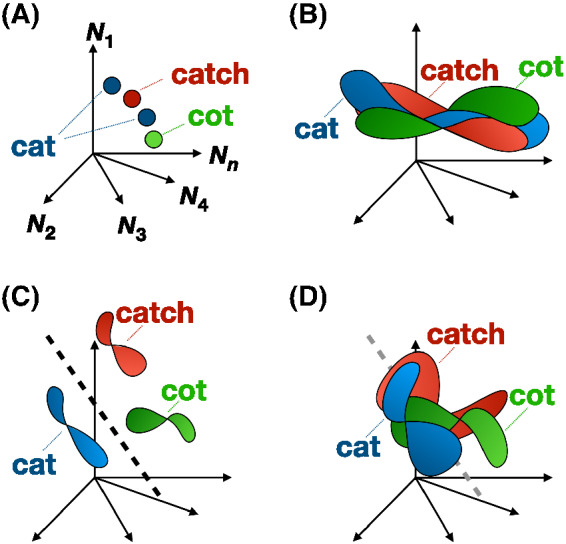
Illustration of Manifold Untangling Across the Auditory and Language Pathways in Typical Development and Developmental Language Disorder (DLD) *Note*. (A) the spoken words *cat*, *catch*, and *cot* in high dimensional space, with each axis (*N*_1_–*N*_*n*_) illustrating the response of a single neuron in a population, in spikes per second. Two spoken instances of the same word, for example, *cat*, will reside in a different neural response vector. (B) collectively, response vectors associated with any given word form a manifold. Manifolds of different words are tangled early in the auditory–linguistic pathway due to cellular responsiveness to low-level acoustic features. (C; a high-capacity system) manifolds are incrementally untangled throughout the auditory pathway, eventually supporting efficient discrimination and reducing attentional demand. (D; a low-capacity system) in developmental language disorder (DLD), a low-level auditory–perceptual deficit may mean that manifold untangling is protracted, leading to abnormally high-dimensional, high-order speech representations that are more difficult to discriminate and which therefore overwhelm attentional capacity. See the online article for the color version of this figure.

The manifold untangling framework has at its heart the notion that acoustic speech signals stimulate patterns of firing in populations of neurons that may be understood as a response vector in high dimensional space; a principle illustrated in Figure 1A (Chung et al., 2018; Cohen et al., 2020; DiCarlo & Cox, 2007; DiCarlo et al., 2012; Stephenson et al., 2020; Yamins & DiCarlo, 2016). Due to speaker variability, co-articulation effects, and background noise, no two instances of any given spoken word are acoustically identical, and so each spoken instance of a given word stimulates a different neural response vector. The collection of neural response vectors associated with any specific word defines that word’s neural manifold.[Fig fig1]

The manifold untangling framework quantifies changes in the dimensionality and separability of manifolds across a processing hierarchy; in our case the auditory–linguistic pathway (Stephenson et al., 2020). Crucial here is the idea that the manifolds underpinning different spoken words are significantly tangled (i.e., intersecting or overlapping) and thus difficult to separate early in the processing stream (Figure 1B). In the cochlea, for instance, this overlap is due to the responsivity of spiral ganglion cells to low-level acoustic features. Neural representations at this level capture variance in the multiple acoustic signals corresponding to any given spoken word, and are, therefore, described as *form dependent* or *noise sensitive*. Transformations instantiated across the typical auditory processing hierarchy result, however, in input-invariant neural responses that are reduced in dimensionality, that is, which are substantiated in patterns of activation across relatively small subspaces of a given neural population, and which are therefore more easily separated from the neural response patterns underpinning competitor classes (Figure 1C). In typically developing individuals, this is demonstrated in increasingly *form independent* or *speech selective* neural responses across the auditory pathway. Acoustic distortion is shown to stimulate the auditory pathway up to and including at the primary auditory cortex (i.e., the core) and the belt, for instance, with increasing speech selectivity, or, by the same token, reduced sensitivity to low-level acoustic features including noise, then observed in the parabelt and more distal substrates (Davis & Johnsrude, 2003; DeWitt & Rauschecker, 2012; Kaas et al., 1999; Okada et al., 2010). This process of transformation defines the central objective of the auditory–linguistic pathway: To establish input-invariant neural speech representations.

The impact of low-level auditory–perceptual deficits on successful manifold untangling (i.e., the shift from form-dependent to form-independent neural responses) is, to our knowledge, as yet unstudied. However, it might be assumed that such auditory–perceptual deficits, which demonstrably characterize the profiles of some children affected by DLD (Bishop & McArthur, 2005; McArthur & Bishop, 2005), would prompt atypical trends in neural response transformation throughout the auditory–linguistic pathway. Specifically, we might expect that the degree of untangling achieved on the basis of degraded speech signals would be lower than the degree of untangling achieved on the basis of clean speech signals. Faced with poor auditory processing ability, neural systems may struggle to reduce manifold dimensionality and establish input-invariance, with low-level noise contaminating high-level speech representations and rendering them highly dispersed. The manifold untangling framework therefore has the potential to shape a precise linking hypothesis from low-level auditory–perceptual deficits to higher-order deficits in speech representation in DLD, while providing a formal description of the latter in terms of neural response manifolds characterized by abnormally high dimensionality.

Furthermore, and fundamental to the primary line of argument pursued in this report, the manifold untangling framework demonstrates how attentional capacity may be overloaded by the low separability of atypically dispersed neural speech representations (Cohen et al., 2020; Stephenson et al., 2020). Recall, for instance, our earlier citation from Oberauer (2019) on the importance of high-quality long-term encodings for the effective deployment of attention. Efficient speech recognition and production depend on rapidly and accurately isolating and retrieving required speech representations from an activated long-term memory cohort, a capacity to which attentional control is central. If we assume that auditory–perceptual deficits do characterize the profiles of some children affected by DLD, and if we can show that these low-level deficits are linked to the formation of higher-order speech representations characterized by amplified levels of dispersion and overlap (i.e., residual manifold tangling), then we might further conclude that the performance profiles commonly attributed to working memory capacity limitations in DLD instead reflect attention being overloaded as a result of long-term speech representations characterized by low discriminability. As we show in the *Method* section (see *Analysis* section), recent computational realizations of the manifold geometry view of neural responses provide the tools required to formally quantify both speech representation dimensionality and associated demands on attentional capacity (Cohen et al., 2020; Stephenson et al., 2020).

## Biological and Artificial Neural Networks

The purpose of the present study is, then, to demonstrate through computational simulations how working memory capacity deficits may emerge as a consequence of atypical speech representation, which itself results from a primary auditory–perceptual deficit. To do this, we use a deep learning framework involving convolutional neural networks, which we describe further in the *Method* section (see *Model* section). State-of-the-art deep learning systems have reached human-level accuracy in speech recognition tasks, and work in computational auditory neuroscience has shown that despite the many substantial differences between biological and artificial neural networks, deep learning can provide valuable insight into human auditory processing and speech representation (e.g., Kell et al., 2018).

There are fundamental parallels between the biological auditory pathway and convolutional network architectures, including the projection of activation into overcomplete space (i.e., activation spreads through layers of an increasing numbers of neurons) and pooling functions (i.e., configurations in which neuron *x* fires if either antecedent neuron *a*, *b*, or *c* fire). The untangling of neural response manifolds is achieved in part as a result of these architectural features, in conjunction with the constraint of response sparseness, that is, top-down pressure on the system to align on a single target representation. As a result of these constraints, the relative size of the subspace in which manifolds reside decreases at each level of transformation, facilitating manifold separability (DiCarlo & Cox, 2007; Kell et al., 2018).

Nevertheless, closer comparisons of the biological auditory pathway and convolutional neural networks, for instance the position that specific artificial layer activation can predict biological auditory–cortical responses (e.g., Kell et al., 2018) remain controversial (Thompson, 2020). One obvious discrepancy between real-world language processing and the simulations presented in the current report is that natural speech signals unfold in time, while processing in a convolutional neural network does not (Stephenson et al., 2020). For our purposes here, then, networks should be understood as providing computational rather than neurobiological insight, in the tradition of Marr (1982), addressing the following questions: What transformation does speech input undergo in order to achieve spoken word recognition? How is this process of transformation impeded due to a low-level auditory processing deficit? And how does any resultant representational abnormality affect demands on attentional control?

In the simulations that follow, we model typical and atypical spoken word recognition by presenting deep convolutional neural networks with spectrograms in which information is either retained to mimic typical development or degraded to mimic the auditory processing deficits identified among some children with DLD (Bishop & McArthur, 2005; McArthur & Bishop, 2005). Computational simulation is essential in enabling us (a) to examine how speech representation differs in artificial neural systems with and without engineered auditory–perceptual deficits and (b) to understand in each case how the form of internal speech representations propagated influences the systems’ ability to retrieve any given representation, a capacity understood as central to attentional control. Crucially, in an artificial system, we are able to ensure that any disparities in network performance are not attributable to an input-independent capacity limitation and are instead attributable exclusively to engineered low-level auditory–perceptual deficits. Our models are not intended to provide a complete picture of speech representation and processing deficits in all children affected by DLD. Instead, we aim to detail a specific causal link previously undescribed in the literature, from auditory–perceptual deficits to speech representation deficits to attentional capacity overload, in the absence of any hard-wired capacity limitation.

## Method

This report is associated with a Jupyter notebook (Kluyver et al., 2016) that can be used to replicate the simulations presented or to experiment with alternative configurations of input, model, and parameters (see https://osf.io/ng6dx/).

### Model

Simulations involved the ResNet-18 convolutional neural network (He et al., 2016), implemented in Python (Python Software Foundation, 2008) using PyTorch (Paszke et al., 2019). A detailed specification of model architecture can be found in the Appendix. For an introduction to convolutional neural networks we recommend Goodfellow et al. (2016; https://www.deeplearningbook.org). In essence, in convolutional layers, these networks pass learned filters over the input, here acoustic spectrograms, in order to identify and summarize through pooling functions invariant features that help solve the task at hand, or, more precisely, that help to reduce output and target discrepancy. For instance, the network might learn that identifying a specific formant pattern captured in a certain distribution of pixels facilitates the discrimination of two phonological competitor words (e.g., *cat*, *catch*), reducing classification error for these items. We trained and tested two populations of networks (*n* = 3) on clean and degraded speech data in a spoken word recognition task. Training lasted for ten epochs (i.e., full cycles through the training data), determined as the point at which networks exposed to clean input approximated 100% accuracy in initial trial simulations involving a restricted dataset.

Crucially, there was no difference in any architectural parameter affecting processing capacity between network populations (e.g., number of layers, hidden layer size, or learning rate). As previously described, the current prevailing view is that fundamental working memory capacity limitations cause speech representation and processing deficits among many children affected by DLD. To reflect this position, a prominent approach in the computational modeling of DLD to date has been to reduce network size, particularly the number of nodes in a network’s hidden layer, explicitly to mimic group differences in working memory capacity (e.g., Takac et al., 2017; Vitevitch & Storkel, 2013). In contrast, in the current report, network processing capacity is reconfigured as an emergent rather than a hard-coded, static, and input-independent parameter, with any performance discrepancies observed between network populations attributable only to access to quality low-level acoustic representations.

### Data

**Figure 2 fig2:**
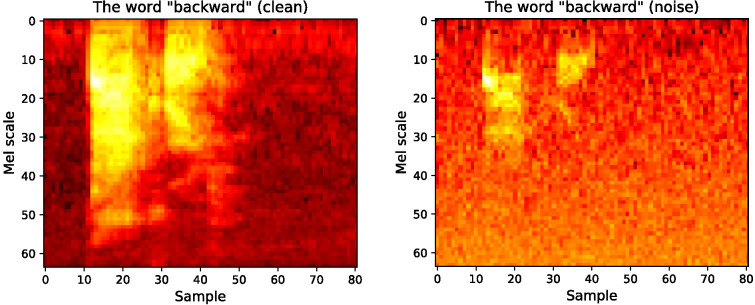
Mel Spectrograms of the Word “Backward,” Clean and With Gaussian Noise (SD = 0.1) *Note*. See the online article for the color version of this figure.

Networks were trained and tested on a random sample of 5,000 instances of spoken words (4,000 training, 1,000 test) from the Speech Commands dataset, which comprises .wav files of different articulations of 35 spoken word types used in the development of keyword recognition systems (e.g., *backward, up, down*, digits 0–9, and a selection of nouns including *bird*, *cat*, and *dog*: see Warden, 2018; see also the Jupyter notebook accompanying the present study). Waveforms were converted to 64-band Mel spectrograms (Stevens et al., 1937), and 0.1 standard deviations of Gaussian noise was added to the training and test data presented to one population of models to simulate the auditory processing deficits observed among some children with DLD (Bishop & McArthur, 2005). The results of this pre-processing can be seen in Figure 2. Our independent variable is, therefore, dichotomous; either a network has access to high quality auditory information, or it does not. In reality, auditory processing ability is likely to be continuous rather than dichotomous in nature, with DLD describing children at the low end of the distribution (see, for instance, Bishop & McArthur’s, 2005, study of individual differences). Nevertheless, our treatment of auditory processing ability as a dichotomous variable represents a welcome simplifying assumption in this first pass analysis of the role of auditory–perceptual deficits on speech representation and working memory in DLD. [Fig fig2]

As we noted in our introduction, the existing evidence suggests that the auditory–perceptual deficits seen among some children with DLD are spectral (i.e., frequency based; e.g., Bishop et al., 1999; Bishop & McArthur, 2005; McArthur & Bishop, 2005) rather than temporal (e.g., Merzenich et al., 1996; Tallal et al., 1996) in nature. Note, however, that the manner in which we add Gaussian noise to spoken word spectrograms in the present study makes it impossible to distinguish between these contrasting accounts. That is, the addition of noise disrupts both frequency information across the vertical axis and temporal information across the horizontal axis (see Figure 2). This is justified because discriminating between the spectral and temporal accounts of auditory processing deficits in DLD is outside of our primary aim to provide an alternative to dominant causal accounts of DLD centered on working memory capacity limitations. With this in mind, we use the general term *auditory–perceptual deficit* (i.e., instead of *frequency processing deficit*) throughout the present study.

### Analysis

Networks were required to identify which word each spectrogram corresponded to by outputting a probability distribution over the 35-word lexicon. The word with the highest assigned probability was considered the network’s selection. As children with DLD often show word finding deficits and response latencies even when making accurate responses (e.g., Messer & Dockrell, 2006), we were interested not only in the networks’ spectrogram classification accuracy, but also in the degree of certainty in accurate classifications made. This required looking not only at the word with the highest assigned probability, but also at the dispersion or *entropy* of the predictive distribution output in response to any given spectrogram. High probability, low entropy predictive distributions reflect greater certainty in a prediction and act as proxy for rapid retrieval, while low probability, high entropy predictive distributions reflect the heightened “consideration” of competitor classes in response to features of the acoustic speech signal presented, and act as proxy for delayed retrieval.

Word classification accuracy and accurate classification predictive distribution probability and entropy are measures of a network’s output. However, crucial to the present study was an assessment of the internal speech representations that networks formed. Manifold dimensionality and classification capacity are variables integral to the computational implementation of the manifold untangling framework, and were estimated following the mean-field-theory based method described in Stephenson et al. (2020) across the networks’ 20 convolutional layers (see Appendix). Readers interested in the mathematical principles via which dimensionality and classification capacity are derived are directed to Cohen et al. (2020) and references therein. In essence, dimensionality quantifies the average degree of dispersion in speech representations across a given neural population (i.e., a network layer), while classification capacity quantifies the network’s average ability to separate any given internal speech representation from competitor representations in a neural population, and therefore provides a measure of demands on attentional control. Algorithmically, dimensionality and classification capacity are determined by propagating activation through the network in order to determine (a) the embedding dimension of the manifold contributing to successful classification (i.e., dimensionality) and (b) the number of word representations that can be linearly separated from competitor representations at each level of the network’s architecture (i.e., classification capacity), standardizing in each case by layer size in order to account for differences in the number of artificial neurons in each layer (Cohen et al., 2020). High classification capacity indicates neural response manifolds having been reduced in dimensionality to facilitate hyperplane separation (i.e., attention is sufficient; Figure 1C), while low classification capacity indicates high-dimensional manifolds unamenable to efficient hyperplane separation (i.e., attention is overloaded; Figure 1D).

Prior research illustrates that dimensionality and classification capacity are not fixed properties (Stephenson et al., 2020). In untrained deep neural networks, little change in manifold dimensionality or classification capacity is seen across layers, from the input layer to the feature layer immediately prior to stimulus classification. In this case, manifolds remain highly dispersed across each layer of the hierarchy, limiting network classification capacity and undermining task performance. However, through training on a specific task, manifold dimensionality decreases across the network hierarchy while classification capacity concurrently increases as a result of improved separability (Chung et al., 2018; Cohen et al., 2020; DiCarlo & Cox, 2007; DiCarlo et al., 2012; Stephenson et al., 2020; Yamins & DiCarlo, 2016). These changes in manifold dimensionality and classification capacity are driven by training and underpin improvements in task performance such as better spoken word classification accuracy.

Through modeling this combination of response variables (i.e., prediction accuracy, probability, and entropy, and manifold dimensionality and classification capacity) as a function input type (i.e., clean versus noisy Mel spectrograms) we were able to analyze both potential variance in performance in a simulated spoken word recognition task and the representation and attentional control factors that underpin that variance. All statistical analyses were conducted in R (R Core Team, 2016; see data repository for analysis script).

## Results

**Figure 3 fig3:**
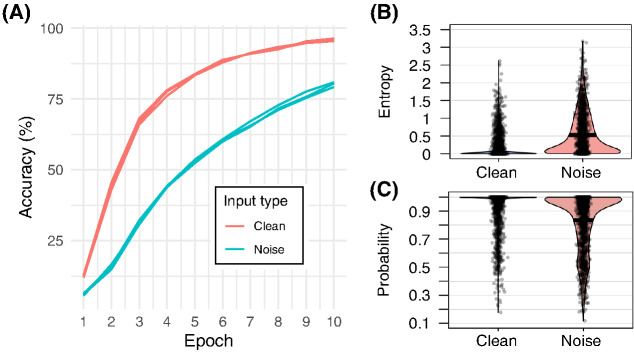
Network Performance During Training and Testing *Note*. (A) accuracy (%) by training epoch and input type. (B) accurate response predictive distribution entropy in bits as a function of input type. (C) probability assigned to accurate predictions as a function of input type. In (B) and (C) black dots represent raw data points, filled portions illustrate densities, and black horizonal bars illustrate means. See the online article for the color version of this figure.

Figure 3A shows training error rates by epoch for each network and input type. Networks exposed to clean input showed a spoken word recognition advantage throughout training, with a mean classification accuracy disparity of 79.9% (*SD* = 2.21) in the clean spectrogram condition, compared to 55.2% (*SD* = 1.59) in the degraded spectrogram condition. Networks exposed to spectrograms that had been degraded by the addition of Gaussian noise not only made fewer accurate predictions, but also showed substantially greater uncertainty in the accurate predictions they made (Figure 3B and 3C). The entropy of accurate predictive distributions generated by networks exposed to clean input was .18 bits (*SD* = .34), with a mean, maximum predictive probability of .94 (*SD* = .13). In contrast, networks exposed to degraded input generated accurate predictive distributions with entropy of .53 bits (*SD* = .59), with a mean maximum predictive probability of .84 (*SD* = .20).[Fig fig3]

**Figure 4 fig4:**
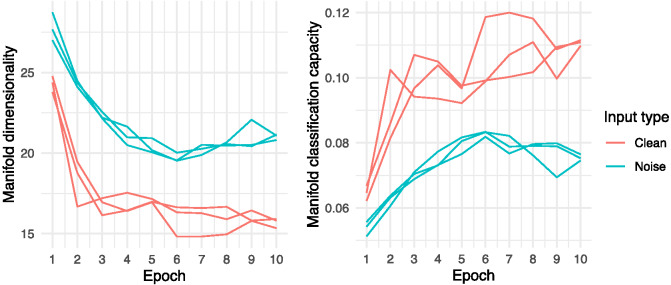
Feature Layer Dimensionality and Classification Capacity by Input Type and Training Epoch *Note*. See the online article for the color version of this figure.

These training and test-phase performance profiles relate directly to the networks’ ability to represent and efficiently retrieve speech information. In Figure 4, we show the average manifold dimensionality and classification capacity during training at the final convolutional layer of each network, immediately prior to the classification layer (see Appendix for network specification). Notably, the divergence in manifold dimensionality between networks presented with clean and degraded input was smaller in relatively early training epochs. Through training, each population of networks reduced the average dimensionality of the internal speech representations it formed in this final convolutional layer. Yet, at asymptote, the divergence between network populations was clear: Reducing the dimensionality of degraded input was an obvious challenge for networks simulating speech representation in DLD. These manifold dimensionality reduction deficits are reflected in the complementary analysis of network classification capacity (Figure 4). Classification capacity increased during training across network populations but was substantially higher in networks modeling typical development. This means that the speech representations formed by the networks modeling typical development were discriminated more easily by a simulated attentional control mechanism than the speech representations formed by the networks modeling DLD, in which attentional control was more rapidly exhausted due to excessive processing demands. In essence, the instantiated auditory–perceptual deficit constituted a significant obstacle to learning, resulting in the formation of spoken word representations that were abnormally dispersed and overlapping (i.e., underpinned by common patterns of neural response), and which therefore could not be easily recognized or retrieved.[Fig fig4]

**Figure 5 fig5:**
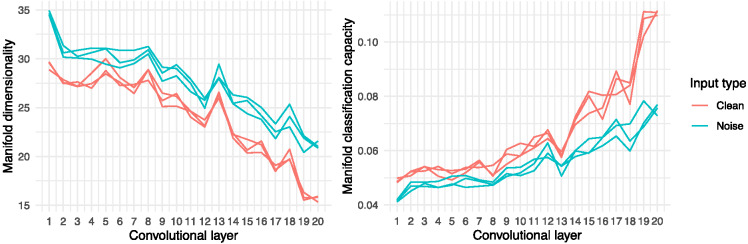
Post-Training Dimensionality and Classification Capacity by Convolutional Layer and Input Type *Note*. See the online article for the color version of this figure.

In Figure 5, a similar trend is shown post training across the networks’ 20 convolutional layers. Neural networks exposed to degraded input never reached levels of manifold dimensionality or classification capacity as low as those seen in the layers of the networks exposed to clean input, and these disparities widened substantially towards the final convolutional layer. Again, networks with engineered auditory–perceptual deficits face a greater challenge in reducing speech representation dimensionality, and this directly impedes the ability of these networks to attend to (i.e., isolate and retrieve) specific internal speech representations. Ultimately, as detailed above, these atypicalities in internal speech representation and simulated attentional control are reflected in disparities in task performance, including reduced speech recognition accuracy and greater uncertainty (i.e., lower probability, higher entropy predictive distributions) even when accurate classifications are made.[Fig fig5]

## Discussion

In this article, our aim has been to provide an alternative to dominant causal accounts of DLD centered on working memory capacity limitations. We developed an account of speech perception, representation, and processing in DLD closely aligned with contemporary working memory frameworks that de-emphasize the role of functionally discrete buffer systems such as the phonological loop in exchange for a more parsimonious characterization of working memory in terms of activated long-term memory plus attention (Adams et al., 2018; Chai et al., 2018; Cowan, 1995; D’Esposito & Postle, 2015; McElree, 2006; Oberauer, 2013, 2019; Wilhelm et al., 2013). We instantiated this theoretical account in a computational model. Simulation demonstrated that protracted manifold untangling provides a plausible link between low-level auditory–perceptual deficits and deficits in higher-order speech representation, as well as a formal description of those speech representation deficits in terms of atypically dispersed patterns of neural response within structures of the auditory–linguistic pathway. This neurocomputational view of speech representation deficits in DLD is broadly consistent with existing verbal descriptions noting the *fuzziness*, *imprecision*, or *indistinctiveness* of these children’s speech representations, and provides a vital counterpart to such accounts (Alt & Suddarth, 2012; Claessen et al., 2009; Claessen & Leitão, 2012; Maillart et al., 2004).

Simulation further illustrated our theoretical view that ostensible shortfalls in working memory capacity may emerge as a consequence of low-level auditory–perceptual deficits propagating neural response manifolds characterized by atypically high dimensionality and residual tangling. Returning to the trade-off described earlier, this suggests that the challenge facing children with DLD may be one of heightened processing demands rather than one of fixed capacity limitations. Children with DLD may be less able to accurately and rapidly process speech sequences and deploy their long-term language knowledge, whether during listening or production, because that long-term knowledge is poorly configured and not amenable to efficiently forming the focus of attention. We showed that representational atypicality (i.e., the heightened dispersion of artificial neural responses) directly undermined the networks’ ability to discriminate any given speech representation within an activated cohort, which is a central function of attentional control. This illustrates how irregularities in long-term speech representation may be the cause of *apparent*, rather than the consequence of *real*, working memory capacity shortfalls. Note that this position differs from the claim that atypical auditory processing restricts the maturation of a working memory buffer system that is functionally discrete from long-term language knowledge (e.g., the *phonological loop*). We posit no such functionally discrete system, and instead attribute a substantial proportion of the variance in working memory task performance to the quality of activated long-term speech encodings. Like prior computational work in this general area (e.g., Jones et al., 2020), the simulations presented here do not provide explicit evidence against a working memory capacity limitation in children with DLD. Rather, they demonstrate a coherent theoretical account of speech perception, representation, and processing deficits in which capacity limitations that are independent of the quality of long-term encodings play no part, and in doing so challenge the status of such limitations as a feature of dominant causal theories of DLD.

Simulation also showed how atypical speech representation and control deficits relate not only to reduced performance accuracy in a spoken word recognition task, but also to substantially greater uncertainty even when making correct responses in that task. Networks with auditory–perceptual deficits made accurate responses characterized by lower maximum probability assignment and higher entropy predictive distributions. This feature of network performance is consistent with behavioral evidence from children with DLD of delays when making accurate responses and associated word finding difficulties, as well as the greater consideration of competitor stimuli in eye-tracking paradigms even when accurate responses are initially made, that is, a child with DLD first orientates accurately to a visual image corresponding to a presented acoustic label (e.g., *net*) but subsequently gazes more regularly at competitor images (e.g., a neck) than age-matched, typically developing control children (Kan & Windsor, 2010; McMurray et al., 2019; Messer & Dockrell, 2006). Regularly, such patterns of performance have been explained by positing auxiliary, encoding-independent processing constraints, for instance generalized slowing (Kail, 1994) or more specific deficits in a hypothesized lateral inhibition mechanism responsible for the successful dampening of activated long-term competitor representations among typically developing children (McMurray et al., 2019). The modeling work presented in the present study suggests, however, that positing constraints that are independent of the quality of long-term speech representations in order to explain such patterns of performance may be unwarranted. Instead, children’s spoken responses may be delayed, or competitor stimuli may be given greater consideration in an eye-tracking paradigm as a result of attention being overloaded by the increased search demands that result from low manifold separability.

Above, we commented against drawing close parallels between the convolutional neural networks used in this study and the biological auditory pathway. However, it is notable that the typically developing brain approximates invariant speech-sound representations by the peripheral auditory cortex (Davis & Johnsrude, 2003), prior to the auditory system splitting into a ventral pathway committed to semantic representation and processing, and a dorsal pathway committed to speech-segment representation and processing, and articulation; each innervated by frontal neural substrates supporting attention (Hickok & Poeppel, 2000). This indicates that approximating invariant speech-sound representations at this juncture is essential to the typical function of the language system as a whole, including to ensuring that attentional resources are not exhausted by uneconomical speech encodings. By the same token, this prior work (e.g., Hickok & Poeppel, 2000) suggests that the protracted manifold untangling simulated in the current report will have wide-reaching implications for the language system as a whole, potentially disrupting the mapping between speech representations and distributed semantics in the ventral stream and speech-segment processing and speech planning in the dorsal stream, as well as disrupting mechanisms of attentional control substantiated in the frontal lobe.

Relatedly, it is valuable to note that prior computational work attests to the generalizability of the principles described in this report. While our own focus has been on auditory perception and the encoding of and attention to spoken word representations, previous research strongly suggests that the auditory–perceptual deficits simulated here would prompt protracted manifold untangling regardless of the level of linguistic representation,that is, whether phoneme, word, or phrase (Stephenson et al., 2020). Indeed, the principles described here are expected to hold regardless of the modality of the stimuli being classified (e.g., whether auditory or visual). There is, therefore, nothing special about words as a unit of representation. Across levels of linguistic representation (i.e., phoneme, word, and phrase), speech recognition and comprehension, retrieval, planning, and production would all be expected to be slower and less accurate as a result of attentional capacity being overloaded by high dimensionality impeding the efficient separation of neural response manifolds. Ultimately, determining the coverage of the theory developed here in explaining the broad constellation of deficits seen in DLD is a matter for future research. There is, of course, no requirement to settle on a single cause of DLD, and indeed such attempts are likely to be fruitless given a complex genetic etiology and the linguistic diversity seen across children with a diagnosis of DLD. Not all children affected by DLD show behavioral deficits or neurophysiological abnormalities in auditory processing (McArthur & Bishop, 2005), and language impairment is not an inevitable consequence of mild to moderate hearing loss (see Halliday et al., 2017, and references therein). Relatedly, there are features of DLD that are not easily reconciled with the notion of a basis in auditory processing deficits. Hsu and Bishop (2014), for instance, report reliable deficits in the ability of children with DLD to identify regular (though difficult to discern) patterns of change in the position of a character on a computer screen (i.e., in a visual serial reaction time task; though, relatedly, see Marshall et al., 2015, for evidence that nonverbal working memory capacity is impacted by language experience). Thus, the manifold untangling deficit hypothesis described in the current manuscript should be considered a complementary explanatory framework, rather than a unifying or absolute theory of DLD.

Attempting to map deficits in manifold untangling to underlying neuronal abnormalities is an important part of the future research agenda. In this report, we situated the locus of deficit at the most fundamental level, the input to the hierarchical processing system. However, given that untangling low-level neural manifolds rests on a protracted and complex hierarchical configuration, including the projection of activation into overcomplete space and pooling functions, it is possible that the problem resides later or more broadly distributed across the auditory pathway, from the basilar membrane to the peripheral auditory cortex, and beyond. Theoretically, unsuccessful manifold untangling may be caused by microneuropathology, in the form of genetic irregularities prompting neuronal mis-migration or inhibiting synaptic pruning, resulting in suboptimal organization within the auditory–linguistic pathway (Bishop, 2014). Future physiological research in this direction might take lead from work assessing neural responses to distorted speech signals in the auditory cortices of typically developing adults (Davis & Johnsrude, 2003; DeWitt & Rauschecker, 2012; Okada et al., 2010). As previously described, this work has identified form-dependent responses to spoken language in the primary auditory cortex and belt, and increasingly form-independent responses in the peripheral auditory cortex and subsequent auditory–linguistic pathways. To our knowledge, it remains unclear whether similar patterns of neural activation across the auditory–linguistic pathway occur in response to different intensities of speech distortion in children with and without DLD.

Given the dominant view that working memory capacity limitations play a causal role in DLD, one line of argument is that interventions specifically targeting working memory can help mitigate these children’s language problems (Delage & Frauenfelder, 2020; Montgomery et al., 2010). As described in our introduction, a number of commercially available programs make this claim (e.g., Alloway et al., 2013). There is, however, little empirical evidence supporting the efficacy of working memory training. For instance, in a comprehensive meta-analysis, Melby-Lervåg and Hulme (2013) found no evidence that apparent gains in working memory function either generalized or remained after a delay period. This outcome is fully continuous with the current report, in which one cause of language impairment is considered to be low-level speech perception and encoding deficits, rather than a functionally discrete working memory capacity bottleneck (see also Jones et al., 2020). Collectively, this work casts doubt on the validity of using working memory training as a method of boosting language skills. As an alternative, simulation showed (across training epochs) that increasing the frequency of exposure to specific structures might go some way to improving long-term encoding and, therefore, to improving the accuracy, speed, and confidence with which long-term speech representations are deployed in the moment. Simulation also suggests, however, that increasing frequency of exposure alone is not enough to effectively close the gap in representation quality and levels of performance between children with and without DLD. In Figure 4, we illustrated clear divergence in dimensionality and classification capacity between network populations at asymptote across ten training epochs (a pattern which may differ under longer training regimes). This suggests that more nuanced strategies than simply boosting frequency of exposure are required in order to mitigate the perceptual and representational challenges faced by children affected by DLD. One such approach, already well-known to clinical practitioners including speech and language therapists, is to control the order of stimulus presentation, for instance by teaching minimal pairs (e.g., *cat*, *catch*) in which the discrepant phoneme is a sound that the child has particular difficulties with (Dean et al., 1995). As high-order neural response manifolds adapt to task and communicative demands through time (Stephenson et al., 2020), this approach is expected to improve the discriminability of the representation of the different constituent and therefore the word-level representation. This view redescribes the computational process highlighted in the *Method* section in which neural networks attune to the specific subpatterns within speech signals that most effectively reduce performance error.

The prior example alludes to the importance of working across levels of linguistic representation during language intervention, here improving spoken word representation (and indeed phrase-level speech representation) by improving sublexical speech segment representation. Ultimately, given the complex causal basis of DLD emphasized earlier, comprehensive programs of intervention that target multiple aspects of the language system appear essential (i.e., because highly specific programs of intervention only focus on remediating a subset of the underlying issues). This factor may explain the limited success of targeted commercial packages of auditory processing intervention such as *Fast ForWord* (Tallal, 2013) in randomized controlled trials (Strong et al., 2011). Relatedly, it would, as one anonymous reviewer pointed out, be wrong to assume that programs of intervention only work if they address an identified area of deficit, as working with an area of relative strength may also help overall language functionality. Along these lines, it is reported that individuals with strong semantic (and syntactic) awareness of the language they are perceiving are better able to decode vocoded elements within a sentence by exploiting top-down predictive processing, in the same manner that the occluded orthographic representation *g##d#n* might be rapidly decoded by exploiting antecdent information in the phrase “it was a sunny day and the children were playing in the *g##d#n*” (i.e., *garden*; Davis et al., 2005; Sohoglu et al., 2012; see Jones & Westermann, 2021, for an application of the predictive processing framework to the study of DLD). While it may be challenging to translate this specific research finding directly into a task to use during language intervention, it is nevertheless valuable to note that strengthening semantic and syntactic awareness may help children with DLD navigate the perceptual and representational deficits that constitute a major obstacle to effective communication.

### Conclusion

In this report we have presented an alternative to dominant theoretical accounts of DLD centered on deficits in working memory capacity. Our account aims to reposition the proximal origin of many of the behavioral deficits seen in DLD from a shortfall in working memory capacity, to working memory being itself functionally unimpaired but overloaded due to operating on speech representations characterized by atypically high dimensionality and low separability.

## Footnotes

1 As Vance (2008) has commented, the terms *working memory* and *short-term memory* are used differently and sometimes interchangeably across studies of DLD. In the present study, working memory refers to both the system supporting the temporary retention of activated long-term representations in response to a perceived stimulus (short-term memory, or the *phonological loop*) *and* the attention system that operates on (i.e., selects, inhibits, or manipulates) those activated representations (attentional control, or the *central executive*).

## ResNet-18 Specification

**Table:**

Layer index	Layer	Output size	Kernel size	Stride
1	2D convolution	1, 64	7, 7	2, 2
2	2D convolution	64, 64	3, 3	1, 1
3	2D convolution	64, 64	3, 3	1, 1
4	2D convolution	64, 64	3, 3	1, 1
5	2D convolution	64, 64	3, 3	1, 1
6	2D convolution	64, 128	3, 3	2, 2
7	2D convolution	128, 128	3, 3	1, 1
8	2D convolution	64, 128	1, 1	2, 2
9	2D convolution	128, 128	3, 3	1, 1
10	2D convolution	128, 128	3, 3	1, 1
11	2D convolution	128, 256	3, 3	2, 2
12	2D convolution	256, 256	3, 3	1, 1
13	2D convolution	128, 256	1, 1	2, 2
14	2D convolution	256, 256	3, 3	1, 1
15	2D convolution	256, 256	3, 3	1, 1
16	2D convolution	256, 512	3, 3	2, 2
17	2D convolution	512, 512	3, 3	1, 1
18	2D convolution	256, 512	1, 1	2, 2
19	2D convolution	512, 512	3, 3	1, 1
20	2D convolution	512, 512	3, 3	1, 1
21	Linear transformation	35	n/a	n/a
Hyperparameters				
Optimizer: Stochastic gradient descent				
Learning rate: .001				
Momentum: .9				
Loss function: Cross-entropy loss				
*Note*. See Jupyter Notebook for activation functions and pooling, normalization, and dropout layers.				
